# Drifting through Basic Subprocesses of Reading: A Hierarchical Diffusion Model Analysis of Age Effects on Visual Word Recognition

**DOI:** 10.3389/fpsyg.2016.01863

**Published:** 2016-11-25

**Authors:** Eva Froehlich, Johanna Liebig, Johannes C. Ziegler, Mario Braun, Ulman Lindenberger, Hauke R. Heekeren, Arthur M. Jacobs

**Affiliations:** ^1^Department of Education and Psychology, Freie Universität BerlinBerlin, Germany; ^2^Dahlem Institute for Neuroimaging of Emotion, Freie Universität BerlinBerlin, Germany; ^3^Center for Cognitive Neuroscience, Freie Universität BerlinBerlin, Germany; ^4^Laboratoire de Psychologie Cognitive, CNRS and Aix-Marseille UniversitéMarseille, France; ^5^Centre for Cognitive Neuroscience, Universität SalzburgSalzburg, Austria; ^6^Max Planck Institute for Human DevelopmentBerlin, Germany

**Keywords:** hierarchical diffusion modeling, aging, letter identification, lexical decision, phonological decision, semantic decision, visual word recognition, reading

## Abstract

Reading is one of the most popular leisure activities and it is routinely performed by most individuals even in old age. Successful reading enables older people to master and actively participate in everyday life and maintain functional independence. Yet, reading comprises a multitude of subprocesses and it is undoubtedly one of the most complex accomplishments of the human brain. Not surprisingly, findings of age-related effects on word recognition and reading have been partly contradictory and are often confined to only one of four central reading subprocesses, i.e., sublexical, orthographic, phonological and lexico-semantic processing. The aim of the present study was therefore to systematically investigate the impact of age on each of these subprocesses. A total of 1,807 participants (young, *N* = 384; old, *N* = 1,423) performed four decision tasks specifically designed to tap one of the subprocesses. To account for the behavioral heterogeneity in older adults, this subsample was split into high and low performing readers. Data were analyzed using a hierarchical diffusion modeling approach, which provides more information than standard response time/accuracy analyses. Taking into account incorrect and correct response times, their distributions and accuracy data, hierarchical diffusion modeling allowed us to differentiate between age-related changes in decision threshold, non-decision time and the speed of information uptake. We observed longer non-decision times for older adults and a more conservative decision threshold. More importantly, high-performing older readers outperformed younger adults at the speed of information uptake in orthographic and lexico-semantic processing, whereas a general age-disadvantage was observed at the sublexical and phonological levels. Low-performing older readers were slowest in information uptake in all four subprocesses. Discussing these results in terms of computational models of word recognition, we propose age-related disadvantages for older readers to be caused by inefficiencies in temporal sampling and activation and/or inhibition processes.

## Introduction

Reading, one of the most complex activities of the human brain, is a life-long learning process (Wolf, 2007; Schrott and Jacobs, 2011), which is performed effortlessly and routinely by most individuals even in old age. Especially for older adults, reading is not only one of the most popular leisure activities, but it is also essential for successfully mastering and participating in everyday life thus contributing significantly to maintaining functional independence (Meyer and Pollard, 2006). Yet, it is still an open issue to what extent age-related changes in perceptual-attentional or higher cognitive processes influence this important daily life activity (Froehlich and Jacobs, 2016). Moreover, considering the multitude of subprocesses underlying visual word recognition and reading, it is of interest to investigate how age affects these subprocesses. For example, if it is the case that vocabulary knowledge increases with age (Verhaeghen, 2003), one might expect beneficial effects of age on lexical processing but such an effect has not been consistently found in the literature (e.g., Allen et al., 1991; Balota and Ferraro, 1996). The aim of the present study was to systematically investigate the impact of aging on four basic subprocesses of reading (sublexical, lexical, phonological, and semantic) in a model-guided way using hierarchical diffusion modeling.

### Hierarchical Diffusion Modeling

A potential reason for the somewhat inconclusive findings of age-related effects on word recognition and reading are the statistical methods used for analyzing age-related differences. Usually, results have been established comparing mean response times (RTs) of younger and older adults in basic decision tasks. The problem of this approach is that older people respond generally slower than younger adults (e.g., Salthouse, 1996), which might mask more subtle processing differences. One approach to circumvent this problem has been the use of Brinley plots, in which mean RTs of older adults are plotted against mean RTs of younger adults. The resulting graph can then be described in mathematical terms with the slope representing the amount of generalized slowing in older adults (Brinley, 1965). However, Brinley plots are of limited use for the assessment of the range of cognitive processes involved in a given decision task, as they neglect informative components of the experimental data, such as correct and incorrect RT distributions or accuracy rates (Ratcliff et al., 2004b).

Addressing this general issue in a more differentiated, model-guided approach, Ratcliff (1978) introduced diffusion models for analyzing two-alternative forced choice tasks. One of the advantages of this approach is that it takes into account a wider set of data, i.e., correct and incorrect RTs, their distributions as well as accuracy data. This facilitates model development by increasing constraints (Grainger and Jacobs, 1996). More important for the present purpose, it allows to disentangle several processing components that underlie performance in a decision task (Ratcliff and Rouder, 1998; Ratcliff et al., 2004c; Ratcliff and McKoon, 2008). A diffusion model conceptualizes the decision process as a continuous sampling of information that accumulates information over time until one of two decision thresholds is reached followed by the actual response. The original model postulates four parameters describing the decision process: a non-decision time (*t*) representing the time needed for stimulus encoding, configuration of task-related working memory, preparation and execution of motor response, the decision threshold (*a*) indicating the amount of information needed for making a decision, the drift rate (*v*), i.e., the speed of evidence accumulation over time reflecting processing efficiency, and the starting point (*z*) that maps potential *a priori* decision biases (Ratcliff, 1978; Voss et al., 2013). In appropriate task contexts, these parameters can directly be interpreted. When motor response preparation requires little effort (e.g., a simple button press), large estimates for *t* are interpreted as increasing difficulties in stimulus encoding. Large estimates for *a* indicate a conservative and slow decision style, while small estimates imply fast but less accurate decisions, thus explaining the commonly observed speed-accuracy tradeoff. Large estimates for *v* are typically a sign of faster information processing (Ratcliff and Smith, 2010; Voss et al., 2013; Oganian et al., 2016).

The diffusion model has been successfully applied to data from a variety of basic decision tasks that are directly or indirectly related to reading, such as letter discrimination (Thapar et al., 2003), lexical decision (Ratcliff et al., 2004a; Oganian et al., 2016), semantic decision (Spaniol et al., 2006; Vandekerckhove et al., 2010), and verbal working memory (Ratcliff, 1978). However, estimating diffusion model parameters requires a large number of data points per participant. With a limited number of trials (*N* < 200; Ratcliff and Tuerlinckx, 2002), parameter estimation might be inaccurate (but see Lerche et al., 2016). Finally, in diffusion modeling, statistical inference is restricted to the specific sample and it does not allow for the investigation of interindividual differences (Vandekerckhove et al., 2011; Voss et al., 2013). To overcome these restrictions, Vandekerckhove et al. (2011) proposed a novel analytical approach combining the advantages of diffusion models and hierarchical models (Gelman and Hill, 2007). Hierarchical diffusion models explicitly allow to estimate diffusion model parameters for individual participants even if the number of data points per subject is relatively small. This makes hierarchical diffusion modeling an ideal method in fields such as psycholinguistics and reading where it is not always possible to generate a large number of stimuli per experimental condition that require careful matching and multiple controls (e.g., Jacobs et al., 2015).

### Reading as a Multicomponent Activity

The complexity of reading may seem astonishing considering the ease with which most individuals perform it in everyday life. Successful reading depends on the interplay of multiple (sub-)conscious cognitive processes. The most basic and central of these processes, visual word recognition (Jacobs and Ziegler, 2015), is typically divided into sublexical (letter recognition, integration of letters into larger sublexical units), orthographic/lexical (whole word recognition), phonological (mapping these letters/units onto sounds) and lexico-semantic processes (assigning meaning to a string of letters; cf. Ziegler et al., 2008). Computational models of reading have successfully tried to capture this complexity by implementing these subprocesses in an interactive activation or parallel distributed processing approach (Grainger and Jacobs, 1996; Plaut et al., 1996; Zorzi et al., 1998; Harm and Seidenberg, 1999; Coltheart et al., 2001; Perry et al., 2007; Hofmann and Jacobs, 2014). Yet, computational models of the full literary experience provided by reading – including affective and aesthetic aspects – still await further development (Jacobs, 2015a,b,c).

At the neural level, numerous brain regions have been identified to be functional in reading allowing to isolate networks that are systematically associated with these four subprocesses (e.g., Price, 2012). While visual and orthographic processes are mainly associated with left posterior inferior occipital as well as left ventral occipitotemporal activations, phonological and semantic processes additionally recruit higher-order language areas, such as left temporal and left inferior frontal regions (e.g., Poldrack et al., 1999; Dehaene et al., 2002; Kronbichler et al., 2007; Welcome and Joanisse, 2012; Schurz et al., 2014; Braun et al., 2015; Cavalli et al., 2016).

### Age-Related Effects on Subprocesses of Reading

It is a still open question how these basic subprocesses and their neural underpinnings are affected by age. A consistent finding concerns age-related deficits at the sublexical level (e.g., word length effects in lexical decision; Froehlich and Jacobs, 2016). Further evidence stems from studies employing letter detection or letter matching tasks in younger and older adults that systematically report RT disadvantages for the older age group (Guttentag and Madden, 1987; Allen et al., 1991; Madden et al., 2007). However, these findings are exclusively based on comparisons of mean RTs or the application of Brinley plots, respectively. Thus, they do not allow us to draw inferences as to whether the observed disadvantages are due to deficits in letter identification/discrimination in old age or whether they are caused by mere (perceptual) decoding difficulties in older adults as has previously been suggested (e.g., Allen et al., 1991, 1993; Aberson and Bouwhuis, 1997). A solution to this problem was offered by Thapar et al. (2003), who used diffusion modeling to examine the effects of aging on letter discrimination. They observed larger estimates for the decision threshold (*a*) for older compared to younger adults, implying a more conservative decision criterion for the older age group. More importantly, estimates for the non-decision time (*t*) were also larger for older participants than for younger ones, whereas the rate of evidence accumulation (*v*) was found to be smaller. Both of these results indicate a slowing in basic encoding processes and a slower uptake of information specific to letter discrimination in older compared to younger adults.

At the orthographic processing level, the lexical decision task is one of the most popular tasks for investigating age-related effects on word recognition and reading. Consistently, in all of the 16 studies recently reviewed by Froehlich and Jacobs (2016), mean RTs of older readers were longer than those of younger ones. However, these findings might have resulted from a mere general age-related slowing in older adults. More importantly, the frequency effect, a standard indicator for the efficiency of lexical access (e.g., Jacobs and Grainger, 1994; Balota et al., 2004) was found to be identical across age groups in 12 of the reviewed studies. This is evidence for preservation of orthographic processing across the life span, as also suggested by results from Cohen-Shikora and Balota (2016) as well as Ratcliff et al. (2004c). The latter applied diffusion modeling to data from a lexical decision task and observed no differences in the rate of evidence accumulation (*v*) between older and younger adults. Yet, similar to letter discrimination, larger estimates were found for the decision threshold (*a*) and the non-decision time (*t*) in older adults compared to younger ones.

At the phonological level, age-related language effects have predominantly been investigated using speech production tasks or tasks employing auditorily presented sounds, words and sentences (cf. Thornton and Light, 2006; Burke and Shafto, 2008). When it comes to age-related effects on phonological processing in visual word recognition, however, there is an apparent lack of research, although it is known that phonological representations are automatically activated during silent reading even in highly skilled readers (e.g., Ziegler and Jacobs, 1995; Braun et al., 2009, 2015; Briesemeister et al., 2009). The phonological decision task, which forces participants to engage in phonological rather than in orthographic processing during silent reading, is therefore exceptionally well suited for our purposes and has not yet been administered to an older cohort (Kronbichler et al., 2007; Bergmann and Wimmer, 2008). There is, however, evidence of age-related slowing in pseudoword reading (e.g., Madden, 1989; Stadtlander, 1995; Bush et al., 2007), which requires successful phonological recoding (e.g., Jacobs et al., 1998; Coltheart et al., 2001; Perry et al., 2007; Taylor et al., 2012).

Finally, to investigate age effects at the lexico-semantic level, several studies have employed semantic decision tasks, in which a target stimulus is classified according to a prespecified category (e.g., Forster and Shen, 1996). Age-related RT effects on semantic processing have been found to be either absent (Daselaar et al., 2003) or larger in older adults (Spaniol et al., 2006). Yet again, analyzing only mean RTs or the accuracy of responses does not allow one to decide whether peripheral encoding processes or semantic processes decline with age. A first step toward solving this issue was made by Spaniol et al. (2006), who applied diffusion model analyses to semantic categorization data of older and younger adults. They found non-decision time (*t*) to be longer in older adults for living versus non-living discrimination. Decision thresholds (*a*) and drift-rates (*v*), however, were comparable for both younger and older participants suggesting a preservation of the speed of lexico-semantic information uptake in age.

In summary, the aforementioned results from diffusion modeling of data from sublexical, orthographic, and lexico-semantic decision tasks point toward non-decision times (*t*) being generally longer in older adults compared to younger ones. The decision threshold (*a*) seems to be more conservative in older than in younger adults in sublexical and orthographic, but not in semantic processing. Age-related effects on the speed of information uptake (*v*) appear to be confined to sublexical processing, whereas orthographic and semantic processing seems to be unaffected by age.

### The Present Study

The aim of the present study was to systematically investigate the impact of age on four basic subprocesses of reading. Use of hierarchical diffusion modeling allowed us to differentiate between age-related influences on decision thresholds, stimulus encoding processes, preparation and execution of motor responses as well as the degree of information uptake during specific reading-related tasks. To illustrate the gain of interpretable information in hierarchical diffusion modeling compared to standard RT analyses, we additionally performed mixed-effects modeling of RTs and accuracy rates. Subprocesses of reading performance were assessed with the help of a letter identification task (visual sublexical processing), a lexical decision task (orthographic processing), a phonological decision task (phonological processing), and a semantic decision task (lexico-semantic processing). We administered these two-forced choice alternative decision tasks to three groups of participants: young adults, high-performing older adults and low-performing older adults. The older reader cohort was split into two groups based on their performance in a sentence comprehension task. This was done because the heterogeneity in cognitive performance increases with age (de Frias et al., 2007; Lindenberger et al., 2008). Considering older adults as a homogenously performing group may leave valuable information undetected. Analyzing these two groups separately might possibly explain the inconsistent findings reported above.

Based on previous evidence, we hypothesize older adults to have prolonged non-decision times (*t*) compared to younger adults in all four decision tasks (Ratcliff et al., 2001, 2003, 2004c,d; Thapar et al., 2003; Spaniol et al., 2006). As non-decision times depend on speed of sensorimotor preparation, perceptual encoding of stimuli, as well as task-related working memory processes, it is expected that older adults show a disadvantage (e.g., Madden et al., 1993; Salthouse, 1996; Vallesi et al., 2009). Likewise, we expect both groups of older adults to show a higher decision threshold (*a*) than younger adults due to a generally more conservative decision strategy (Ratcliff et al., 2001, 2003, 2004c,d; Thapar et al., 2003 but see Spaniol et al., 2006, Experiment 1).

The major interest of the present study concerns age-related effects on the speed of information uptake during sublexical, orthographic, phonological, and semantic processing. At the sublexical level, we expected lower drift rates (*v*) in the older group than in younger readers (cf. Thapar et al., 2003). In contrast, the speed of information uptake was thought to be unaffected by age in orthographic and lexico-semantic processing (cf. Ratcliff et al., 2004c; Spaniol et al., 2006). Concerning phonological processing, we can only speculate about the outcome as (to our knowledge) this is the first study to explicitly investigate phonological processing during visual word recognition in aging. Based on findings from pseudoword reading in lexical decision, we assume an age-disadvantage for the older group (Madden, 1989; Stadtlander, 1995; Bush et al., 2007), especially when considering the amount of focused spatial attention phonological processing requires (cf. Facoetti et al., 2006). With the additional grouping of the older cohort, we expect to gain a more differentiated and informative picture of the effects of aging on the four central subprocesses of reading with respect to all three hierarchical diffusion modeling parameters.

## Materials and Methods

### Participants

The present study recruited a subsample of 1,807 subjects (930 female, 877 male) from the Berlin Aging Study II cohort (BASE-II; Bertram et al., 2014). Selection was based upon completion of all reading tasks with individual error rates below 40%. The 384 younger adults (195 female, 189 male) were on average 30.7 years old (range 23–40 years). The sample of the 1,423 older adults (735 female, 688 male) was further split into two groups based on their performance in a sentence comprehension task. Using the SOS-algorithm (Armstrong et al., 2012), we identified 384 older adults (191 female, 193 male) whose reading scores were on average identical to that of younger participants (*M* = 60.7) and 1,039 older participants (544 female, 495 male) who differed significantly in their performance from the other two groups (*M* = 52.0, *p* < 0.001). High-performing older adults were on average 69.7 years old (range 61–88), low-performing older adults 70.2 years (range 60–86). The age difference between the older groups was not significant (*p* = 0.11, Tukey corrected). Due to technical problems during data acquisition information on education could not be evaluated for 14.7% of the participants (young adults = 19.5%, high-performing older adults = 10.7%, low-performing older adults = 15.3%). The remaining participants differed in years of education with low-performing older participants having less years of education (*M* = 14.1) than high-performing older participants (*M* = 14.6; *p* < 0.05) and younger participants (*M* = 15.0; *p* < 0.001). The two latter groups did not differ from each other (*p* = 0.09). All participants were German native speakers, right-handed and had normal or to normal corrected vision. None of the participants had a history of reading difficulties or language impairment, neurological disease, psychiatric disorders or a history of head injuries. Prior to the study, written informed consent was obtained and subjects received financial compensation for their participation. The study was approved by the Ethics Committee of the Max Planck Institute for Human Development, Berlin (MPIB).

### Procedure

Participants were tested in small groups of up to six individuals in a quiet test room on the Charité Campus, Berlin. One test session lasted for about 3.5 h and included additional tasks of the BASE-II test battery. Before each task, participants performed training trials. The following tasks were used to assess the four central subprocesses of reading: letter identification, lexical, phonological, and semantic processing. The order of the tasks was as follows: Sentence comprehension task, phonological decision task, semantic decision task, lexical decision task, letter identification task. Within each task, item order was pseudorandomized and items were presented one by one at the center of a computer screen for 3 s or up to participant’s response. Participants were instructed to give yes- or no-responses via button press as quickly and accurately as possible.

### Stimuli and Design

To ensure comparability of results and control for confounding linguistic variables, item length, number of orthographic neighbors, bigram frequency and (base word) frequency were carefully matched across the lexical, the phonological as well as the semantic decision task (all *F*’s < 1.83, all *p*’s > 0.2). Within these tasks, the mean number of letters per item varied from 4.43 to 4.53, the mean number of orthographic neighbors from 19.4 to 24.4. Normalized lemma frequency of the (base) word frequency ranged from 1.22 to 1.41, the bigram frequencies from 4.35 to 4.42. Bigram frequencies in the letter identification task were lower than those in the other tasks, *F*(3,316) = 33.4, *p* < 0.001, as vowel-consonant combinations were excluded by design. Each item in the letter identification task consisted of five consonants (see Supplementary Table S1 for detailed item characteristics). Matching was based on the dlex database (dlexDB; Heister et al., 2011) norms for German words. Within each task 40 items served as targets and 40 as non-targets.

#### Letter Identification Task

To assess position-specific letter processing without lexical activation (Ziegler et al., 2008), participants had to indicate whether the letter ‘r’ occurred within a consonant string (target; e.g., dbnrl) or not (non-target; e.g., djptd). Targets and non-targets were carefully matched for bigram frequency and had on average the same number of lowercase letters, letters with ascenders and letters with descenders.

#### Lexical Decision Task

Orthographic processing was assessed by presenting either German nouns (targets; e.g., Park) or German pseudohomophones (non-targets; e.g., Waal [whale]). Pseudohomophones are a particular type of pseudowords, but different to pseudowords, which are pronounceable words with no meaning, pseudohomophones sound like real words (e.g., brane is phonologically identical to the real word brain). Participants had to decide whether the presented stimulus was a correctly spelled German word or not. Pseudohomophones were created by changing one letter of an existing German noun to keep them orthographically similar to real words. The initial letters of all items were capitalized to ensure the typical appearance of German nouns.

#### Phonological Decision Task

To investigate phonological processing participants had to judge whether pseudohomophones (target; e.g., Waal) or pseudowords (non-target; e.g., Lase) were presented. Specifically, they were asked to give a yes-response when the item on the screen *sounded* like a word and to give a no-response otherwise. Identical to pseudohomophone construction, pseudowords were created by changing one letter from an existing German noun and were presented with capitalized initial letters.

#### Semantic Decision Task

This task was designed to measure participants’ abilities in lexico-semantic processing. Subjects had to indicate whether the presented item described living objects (target; e.g., Koala) or non-living objects (non-target; e.g., Plan). In line with the previous tasks, only German nouns served as targets and non-targets.

#### Sentence Comprehension Task

Overall reading ability was assessed using a computerized version of a standard German sentence reading test (Bergmann and Wimmer, 2008). Participants had to judge via button press whether each of a total of 77 successively presented sentences was meaningful or not. Sentences gradually increased in sentence length as well as word and morpho-syntactic complexity but were generally easy to comprehend [e.g., Ein Nashorn ist ein Blechblasinstrument (A rhinoceros is a brass instrument)]. Overall reading performance was calculated by summing up the correctly answered items within 3 min. The scores of this task were solely used to differentiate between high-performing older adults and low-performing older adults.

### Data Analysis

#### Outlier Exclusion

Prior to analyses, RTs smaller than 300 ms were excluded to prevent fast guesses from biasing results (Voss et al., 2013). For RT and hierarchical diffusion modeling analyses we then removed RTs deviating more than 2.5 standard deviations from the individual’s mean within each task × stimulus type experimental cell. These procedures led to a removal of 3.37% of the data.

#### Analyses of Mean Response Times and Accuracy

Mean RTs and accuracy were analyzed to illustrate the added value of information the diffusion modeling approach provides. We used mixed-effects modeling (Baayen et al., 2008) as implemented in the “lme4”-package (Bates et al., 2014) with crossed random factors for subjects and items. Analyses were run in R version 3.3.0 (R Core Team, 2016). We analyzed RTs by linear mixed-effects regression, including main effects and interactions for task and age as fixed factors. Fixed effects were tested for significance using Type III Wald chi-square tests (“car”-package; Fox and Weisberg, 2011). Accuracy was analyzed using logistic mixed-effects regression. As recommended by Barr et al. (2013; Barr, 2013), the random factor structure included intercepts for subjects and items, and random slopes for age within items, as well as random slopes for task within subjects.

#### Hierarchical Diffusion Modeling

Response times and accuracies were fitted to hierarchical diffusion models using the python toolbox HDDM, which provides hierarchical Bayesian parameter estimation of the drift-diffusion model (Wiecki et al., 2013). The hierarchical approach allows for simultaneous estimation of diffusion model parameters across participants and the possibility to restrain these parameters according to theoretical assumptions: While some parameters may vary from individual to individual, others are constrained to be equal across participants (Vandekerckhove et al., 2011; Voss et al., 2013). For our purposes, we created a model for each of the subprocesses of reading (i.e., letter identification, orthographic processing, phonological processing, lexico-semantic processing). Within these four models, the non-decision time (*t*), the decision threshold (*a*) with the upper threshold being the correct response and the lower threshold being the incorrect response, as well as the drift rate (*v*) were estimated for each individual separately. These parameters were allowed to vary across participants to account for the general increase in non-decision time (*t*) of older compared to younger participants (Ratcliff, 2008), for individual and age-related preferences in setting the decision threshold (*a*), and, most importantly, for age-effects on the speed of information uptake (*v*) within the subprocesses of reading. We constrained the bias parameter (*z*) of each individual to 0.5 as we did not assume an *a priori* preference of participants toward one of the response options as the number of targets and non-targets were equal. Additionally, following the approach of Oganian et al. (2016), we set parameters of trial-to-trial variances of non-decision time, drift rate and bias parameter to 0. Fixing these parameters can improve parameter estimation of *t, a*, and *v* (Lerche and Voss, 2016). In summary, we estimated the posterior distributions of a total of 36 parameters across the four decision tasks: 12 non-decision time parameters (*t*), 12 threshold parameters (*a*), and 12 drift rate parameters (*v;* for *t, a* and *v* one for each age group within each of the four subprocesses of reading).

Parameters were estimated using a Bayesian approach as implemented in the HDDM toolbox. The Bayesian approach is particularly well suited for hierarchical model estimation (Vandekerckhove et al., 2011). It assigns prior probability distributions to each of the parameters to be estimated and applying the Bayes’ theorem allows the estimation of the posterior probability distribution of the parameters given the observed data. Approximation of posterior distribution is done using an iterative procedure, the Markov chain Monte-Carlo sampling (MCMC; for an introduction to MCMC and Bayesian statistics, see Kruschke, 2015). When running several MCMC chains it is important to ensure that all chains of the model properly converge. We therefore assessed model convergence by visually inspecting the traces of the posterior distribution and in a second step by calculating the Gelman-Rubin statistic (R-hat; Gelman and Rubin, 1992). Comparing the within-chain and between-chain variance of different runs of the same model, this statistic will be close to one if the chains converged successfully. Values exceeding 1.02 indicate problems with convergence (Wiecki et al., 2013) and consequently deficient model estimation.

For each of the four models representing the basic subprocesses of reading, all model parameters were estimated using three MCMC chains with starting values being set to the maximum posterior (as implemented in the HDDM toolbox). The chains contained 15.000 samples drawn from the posterior distribution from which the first 5.000 samples were discarded as burn-in to ensure stabilization of the chains. After controlling for proper convergence, we assessed the quality of model-to-data fit by simulating 500 data sets (RTs and accuracy) from each participant’s model and compared the means of these data sets with the empirical data.

#### Hypothesis Testing

To examine age-related differences in letter identification, orthographic, phonological and lexico-semantic processing on non-decision time (*t*), decision threshold (*a*), and drift rate (*v*), we used Bayesian hypothesis testing as implemented in the HDDM toolbox. For each of the models, we calculated the proportion of overlap of the posterior distributions of the three age groups with respect to the parameters *t, a*, and *v*.

## Results

### Regression Analyses of Response Times and Accuracy

#### Response Times

Mean RTs are shown in **Table 1**. The 4 × 3 (task: letter identification vs. lexical decision vs. phonological decision vs. semantic decision × age: young vs. high-performing old vs. low-performing old) linear mixed-effect model yielded a main effect of task, χ^2^(3) = 867.0, *p* < 0.001 and age, χ^2^(2) = 499.3, *p* < 0.001, as well as the significant interaction of both factors, χ^2^(6) = 41.0, *p* < 0.001. Planned comparisons, directly encoded in the model, showed that shortest RTs were obtained in the letter identification task, *b* = –210.1, *SE* = 14.3, *t* = –14.7. RTs in the semantic decision task were shorter, *b* = –200.7, *SE* = 13.5, *t* = –14.9 than in the lexical and phonological decision task and again RTs in the lexical decision task were shorter than in the phonological decision task, *b* = –252.9, *SE* = 11.8, *t* = –21.4. Generally, younger participants responded faster than older adults, *b* = –105.1, *SE* = 5.38, *t* = –19.5, and high-performing older adults responded faster than low-performing older adults, *b* = –26.3, *SE* = 3.79, *t* = –6.94. The identical age-related RT pattern was observed within all four tasks (see **Table 2** for a detailed summary). Given the size of the data set and all absolute *t*-values being well above 2, we consider these differences to be significant (cf. Baayen et al., 2008).

**Table 1:** Mean RTs (msec), accuracy (%) and standard deviations (*SD*) for all single item reading tasks as a function of age.

	Younger adults	High-performing older adults	Low-performing older adults
**RTs (*SD*)**			
Letter identification task	599 (132)	752 (176)	785 (194)
Lexical decision task	734 (233)	845 (251)	919 (305)
Phonological decision task	1,217 (449)	1,353 (479)	1,407 (490)
Semantic decision task	698 (174)	812 (198)	856 (224)
**Accuracy (*SD*)**			
Letter identification task	97.5 (15.7)	97.9 (14.5)	97.7 (14.9)
Lexical decision task	96.0 (19.6)	97.8 (14.7)	97.0 (17.0)
Phonological decision task	90.2 (29.8)	88.8 (31.5)	87.9 (32.7)
Semantic decision task	96.3 (18.9)	97.6 (15.4)	96.8 (17.6)

**Table 2:** Summary of linear mixed-effect regressions for RTs within the four single item reading tasks.

Predictor	*b*	*SE*	*t*-value
**Letter identification task**			
Intercept	713.4	6.78	105.3
Age^1^	–113.7	4.95	–23.0
Age^2^	–16.6	3.28	–5.07
**Lexical decision task**			
Intercept	840.9	13.4	62.9
Age^1^	–97.8	7.41	–13.2
Age^2^	–38.7	4.99	–7.77
**Phonological decision task**			
Intercept	1,346.7	28.7	46.9
Age^1^	–117.1	11.1	–10.6
Age^2^	–27.3	7.07	–3.86
**Semantic decision task**			
Intercept	792.7	9.28	85.4
Age^1^	–91.6	5.49	–16.7
Age^2^	–22.8	3.39	–6.71

*b, beta-estimate; SE, standard error;*^1^*young adults compared to high- and low-performing older adults;*^2^*high-performing older adults compared to low-performing older adults. Within each task, the main effect of age was highly significant, χ^2^_letter identification_(2) = 598.0, p < 0.001, χ^2^_lexical decision_(2) = 256.6, p < 0.001, χ^2^_phonological decision_(2) = 147.1, p < 0.001, χ^2^_semantic decision_(2) = 346.5, p < 0.001.*

#### Accuracy

Mean percentage accuracy rates are shown in **Table 1**. The 4 × 3 (task: letter identification vs. lexical decision vs. phonological decision vs. semantic decision × age: young vs. high-performing old vs. low-performing old) logistic mixed-effects analyses showed a main effect of task χ^2^(3) = 148.6, *p* < 0.001 and age χ^2^(2) = 19.4, *p* < 0.001 as well as the significant interaction of task and age, χ^2^(6) = 29.7, *p* < 0.001. Highest accuracy rates were obtained in the letter identification task, *b* = 0.43, *SE* = 0.12, *z* = 3.62. Participants made fewer errors in the lexical decision task, *b* = 0.71, *SE* = 0.11, *z* = 6.36 than in the semantic and phonological decision task and fewer errors in the semantic decision task, *b* = 0.93, *SE* = 0.10, *z* = 9.68 than in the phonological decision task. Across all four tasks high-performing older adults showed higher accuracy rates than younger and low-performing older adults, *b* = 0.13, *SE* = 0.03, *z* = 4.05. The same age-related effects were found within the letter identification, the lexical and semantic decision task while within the phonological decision task higher accuracy rates were observed for younger participants than for high-performing older participants and higher accuracy rates for high-performing older participants than for low-performing older participants (see **Table 3** for a detailed summary).

**Table 3:** Summary of logistic mixed-effect regressions for accuracy within the four single item reading tasks.

Predictor		*b*	*SE*	*z*-value	*p*-value
**Letter identification task**					
Intercept		4.43	0.12	37.5	<0.001
Age^1^		0.10	0.04	2.44	<0.05
Age^2^		0.05	0.04	1.47	0.14
**Lexical decision task**					
Intercept		4.57	0.14	31.8	<0.001
Age^1^		0.23	0.06	3.77	<0.001
Age^2^		0.05	0.06	0.75	0.45
**Phonological decision task**					
Intercept		2.66	0.11	23.7	<0.001
Age^3^		0.18	0.06	2.80	<0.01
Age^4^		0.08	0.03	2.70	<0.01
**Semantic decision task**					
Intercept		4.38	0.13	33.2	<0.001
Age^1^		0.29	0.06	5.17	<0.001
Age^2^		0.09	0.05	1.67	0.10

*b, beta-estimate; SE, standard error;*^1^*high-performing older adults compared to young and low-performing older adults;*^2^*low-performing older adults compared to young adults;*^3^*young adults compared to high- and low-performing older adults;*^4^*high-performing older adults compared to low-performing older adults. The main effect of age was significant within each of the tasks, χ^2^_letteridentification_(2) = 6.35, p < 0.05, χ^2^_lexicaldecision_(2) = 17.9, p < 0.001, χ^2^_phonologicaldecision_(2) = 16.8, p < 0.001, χ^2^_semanticdecision_(2) = 27.8, p < 0.001.*

In summary, analyses of mean RTs and accuracy rates suggest an age-related slowing within all subprocesses of reading. Higher accuracy rates were observed for younger participants compared to older participants only within the phonological decision task. For letter identification, lexical decision, as well as semantic decision, high-performing older participants obtained higher accuracy rates than the other two groups. Together with the finding that older adults responded more slowly, this finding suggests a speed-accuracy tradeoff for high-performing older adults compared to younger participants in these three tasks.

### Hierarchical Diffusion Modeling

#### Assessment of Convergence and Model Fit

**FIGURE 1:**
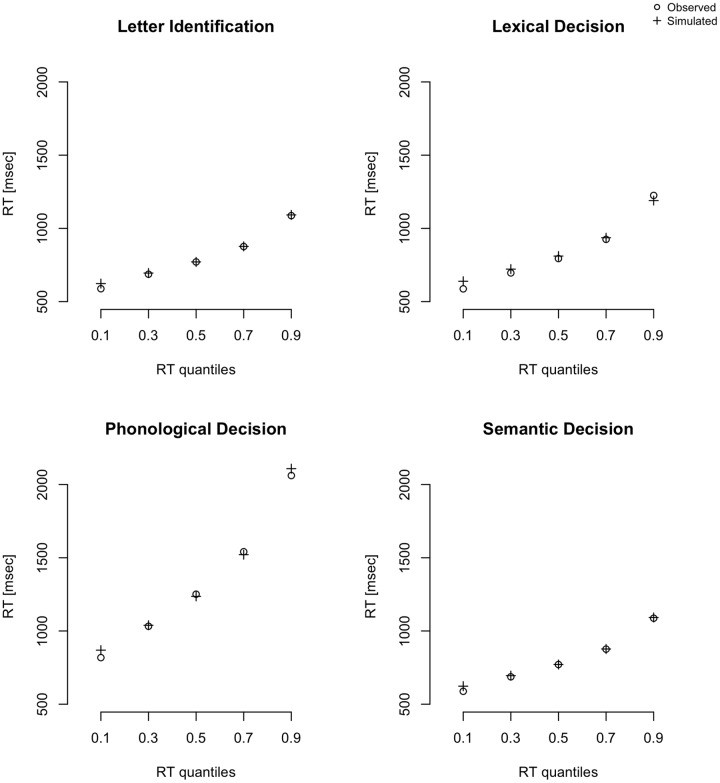
**Plot of RT quantiles (0.1, 0.3, 0.5, 0.7, and 0.9) for correct responses based on observed and simulated data; observed data is within a 95% credibility interval of simulated data**.

Model convergence was assessed by visually inspecting the traces of the posterior distributions and by calculating the *R*-hat statistics for each of the models separately. We neither observed any drifts or large jumps within the plots nor any parameter values above 1.02 within the *R*-hat statistic, indicating successful convergence for all models of reading subcomponents. We then compared the simulated with the observed data, again for all models separately. The model fitted our data very well: The correlation between empirical data and model RT quantiles was *r* = 0.98 in the letter identification task, *r* = 0.94 in the lexical decision task, *r* = 0.99 in the phonological decision task, and *r* = 0.96 in the semantic decision task (**Figure 1**).

#### Model Parameter Analysis of Posterior Estimates

**FIGURE 2:**
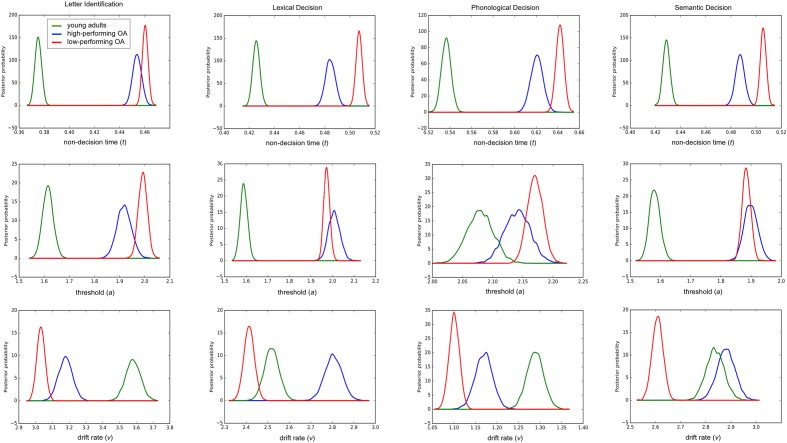
**Posterior density plots of group means of the parameters non-decision time *t* (first line), decision threshold *a* (second line) and drift rate *v* (third line); OA, older adults**.

Analyses of the posterior estimates showed age-related differences in non-decision time (*t*), decision threshold (*a*), and drift rate (*v*) for all four reading tasks (**Table 4**; **Figure 2**).

**Table 4:** Mean posterior estimates for non-decision time (*t*), decision threshold (*a*) and drift rate (*v*) as well as 95% credibility intervals [lower boundary; upper boundary] as a function of age for all single item reading tasks.

	Younger adults	High-performing older adults	Low-performing older adults
**Letter identification task**			
Non-decision time (*t*)	0.376 [0.371; 0.381]	0.453 [0.447; 0.460]	0.459 [0.455; 0.464]
Decision threshold (*a*)	1.62 [1.58; 1.66]	1.92 [1.86; 1.97]	1.99 [1.95; 2.02]
Drift rate (*v*)	3.58 [3.49; 3.66]	3.18 [3.10; 3.26]	3.04 [2.99; 3.09]
**Lexical decision task**			
Non-decision time (*t*)	0.426 [0.421; 0.432]	0.484 [0.477; 0.491]	0.506 [0.502; 0.511]
Decision threshold (*a*)	1.59 [1.56; 1.63]	2.00 [1.95; 2.06]	1.97 [1.95; 2.00]
Drift rate (*v*)	2.52 [2.46; 2.59]	2.80 [2.72; 2.88]	2.42 [2.37; 2.47]
**Phonological decision task**			
Non-decision time (*t*)	0.537 [0.529; 0.546]	0.620 [0.609; 0.631]	0.641 [0.634; 0.648]
Decision threshold (*a*)	2.08 [2.04; 2.12]	2.14 [2.10; 2.18]	2.17 [2.14; 2.20]
Drift rate (*v*)	1.29 [1.25; 1.33]	1.17 [1.13; 1.21]	1.10 [1.08; 1.13]
**Semantic decision task**			
Non-decision time (*t*)	0.430 [0.424; 0.435]	0.487 [0.480; 0.494]	0.505 [0.501; 0.509]
Decision threshold (*a*)	1.59 [1.55; 1.62]	1.90 [1.86; 1.94]	1.88 [1.86; 1.91]
Drift rate (*v*)	2.84 [2.77; 2.91]	2.88 [2.81; 2.95]	2.61 [2.57; 2.65]

As expected, older participants obtained larger estimates for both non-decision time (*t*) and decision threshold (*a*) than did younger participants with a probability ranging from 0.98 to 1 for both parameters for all four tasks as assessed via Bayesian hypothesis testing. Estimates of the non-decision time (*t*) in all four tasks were larger for low performing older adults compared to high-performing older adults with probabilities ranging from 0.94 to 1. Likewise, for the decision threshold (*a*), estimates were larger for low-performing older adults than for high-performing older adults in the letter identification and the phonological decision task with a probability of 0.98 and 0.88, respectively, whereas the opposite pattern was found for the lexical and semantic decision task with a probability of 0.88 and 0.71. Also, as expected, with a probability of 1, drift rates (*v*) were higher for younger than for older adults in the letter identification and phonological decision task. Yet, high-performing older adults obtained higher drift rates than low-performing older adults in both of these tasks, the probability being 1 in both cases. Against our expectations, we found an age-related advantage in drift rates (*v*) for high-performing older adults over young adults within the lexical and semantic decision task (probability of 1 and 0.80, respectively). Low-performing older adults, however, showed an age-related disadvantage compared to younger adults with probabilities of 1 in both tasks.

## Discussion

The present study aimed at systematically investigating age-related effects on four basic subprocesses of reading. To account for the somewhat inconsistent age-related findings in sublexical, orthographic, phonological and lexico-semantic processing reported in the past, we differentiated between high- and low-performing older adults and used a hierarchical diffusion modeling approach. Results based on this approach showed that in general older readers obtained larger estimates for the non-decision times (*t*) and needed more information to make a decision (*a*). Most importantly, though, a different picture emerged with respect to the speed of information uptake within the subprocesses (*v*).

Non-decision times (*t*) were longest for low-performing older adults and longer for high-performing older adults than for younger adults. While high-performing older adults showed higher estimates for the decision threshold (*a*) in orthographic and lexico-semantic processing than did low performing older adults, the opposite pattern emerged for sublexical and phonological processing. Of special interest was the speed of information uptake (*v*). Here, an age-advantage was found for sublexical and phonological processing for young adults compared to high-performing older adults and an advantage was found for high-performing older adults over low-performing older adults. Most importantly, drift rates (*v*) of high-performing older adults in lexical and semantic decision tasks were superior to those of young adults while drift rates of young adults exceeded those of low-performing older adults.

### Age-Related Effects on Response Times and Accuracy

For mean RTs, we replicated the classical finding of an age-related slowing in older compared to younger adults in all four decision tasks. Additional analyses showed that young participants obtained shorter RTs than high-performing older adults and high-performing older adults obtained shorter RTs than did low-performing older adults. Based on these findings, one would have to conclude that all four central subprocesses of reading decline with increasing age to different degrees.

Accuracy rates across all four subprocesses of reading were higher for high-performing older adults than young and low-performing older adults. However, in phonological processing, younger adults obtained higher accuracy rates compared to high-performing older adults and high-performing older adults obtained higher accuracy rates than did low-performing older adults. Taking also mean RTs for this task into account, apparently, phonological processing was most demanding for older adults. Yet in sublexical, lexical and semantic processing, high-performing older adults showed higher accuracy rates than did young and low-performing older adults. In traditional mean RT/accuracy analyses, these results would be hard to interpret since high-performing older adults showed the classical speed-accuracy tradeoff compared to younger adults often reported for older adults in general (e.g., Forstmann et al., 2011; Heitz, 2014).

### Hierarchical Diffusion Modeling

Hierarchical diffusion modeling is superior to traditional mean RT/accuracy analyses as it allows to disentangle a range of cognitive processes involved in decision making such as the time needed to prepare a response (*t*), the amount of information needed to make a decision (*a*), and the speed with which information is accumulated to reach a decision (*v*).

As expected, posterior estimates for non-decision times (*t*) were smaller for young adults than for older adults with an advantage in non-decision time for high-performing older adults compared to low-performing older adults. Yet, it is challenging to draw direct conclusions from these results. The parameter *t* is thought to estimate the time needed to encode the stimulus, to prepare the appropriate motor response, and to configure task-related working memory. However, all of these processes should yield a natural advantage for younger over older adults. Thus, this “compound” parameter still seems too fuzzy to determine the individual contribution of age on each of these subprocesses to allow inferences of age-related slowing on non-decision time in the present study. Further studies specifically targeting the subprocesses contributing to parameter *t* at the behavioral or neural level are strongly needed to clarify this open issue.

Consistently larger estimates for decision threshold (*a*) in all four two-alternative forced choice tasks suggest that older adults in the present study applied a more conservative criterion than younger adults. These results are in line with previous evidence from diffusion modeling studies on aging in various domains (e.g., Thapar et al., 2003; Ratcliff et al., 2004c). Obviously, older adults tend to collect more information before making a decision preferring accuracy over speed than younger adults (e.g., Forstmann et al., 2011). However, while low-performing older adults obtained larger estimates for the decision threshold (*a*) at the sublexical and phonological level than high-performing older adults, the opposite pattern was found at the orthographic and lexico-semantic processing level. Within the diffusion modeling framework, the above mentioned speed-accuracy tradeoff in high-performing older adults at the latter two processing levels can nicely be explained when considering both the decision threshold (*a*) and the drift rates (*v*): Low-performing older adults need to lower their decision threshold (*a*) due to the observed slowing in the speed of information uptake (*v*) to be able to still reach a decision within the designated time window. In lowering their decision threshold (*a*), low-performing older adults are prone to make more erroneous responses (cf. Heitz, 2014), though, a result we observed in comparison to high-performing older adults. Similarly, young adults with the lowest decision threshold (*a*) have lower accuracies than high-performing older adults; yet with a higher speed of information uptake (*v*) than low performing older adults they can afford to set such a liberal decision threshold (*a*). The large drift rate (*v*) observed for high-performing older adults allows this group to settle upon a very conservative decision threshold (*a*) and still make decisions on time with very high precision.

The major focus of the present study was to investigate age-related effects on four basic subprocesses of reading: sublexical, orthographic, phonological and lexico-semantic processes. The efficiency of these subprocesses was analyzed via hierarchical diffusion modeling by the speed of information uptake, i.e., drift rate (*v*). At the sublexical level, we found smaller drift rates (*v*) for low-performing older adults compared to high-performing older adults and, in turn, smaller drift rates (*v*) for high-performing older readers compared to younger readers. This replicates evidence from both classic mean RT analyses and diffusion modeling studies (e.g., Allen et al., 1991, 1993; Thapar et al., 2003; Froehlich and Jacobs, 2016). It is still a matter of debate whether disadvantages for older adults in sublexical processing are mainly the result of difficulties in stimulus encoding or caused by an age-related decline in lexical subprocessing *per se* (e.g., Allen et al., 1991; Aberson and Bouwhuis, 1997). By applying hierarchical diffusion modeling, our results point toward both of these interpretations when considering the larger posterior estimates for non-decision time (*t*) together with the smaller drift rate (*v*) observed for older adults compared to young ones. Larger estimates for *t* have been interpreted as increased difficulties in stimulus encoding, when motor response preparation requires little effort (cf. Oganian et al., 2016), as was the case in the present study. Smaller estimates for the drift rate (*v*) suggest that older adults are also prone to a decline in sublexical processing itself. Yet, the age-related decrease in sublexical processing varied substantially within older subjects of this study. Successful sublexical processing is predominantly based on efficient grapheme to phoneme translation and formation of meaningful letter combinations (e.g., prefixes, syllables) and is thought to be the most basic stage of visual word recognition and reading (e.g., Grainger and Jacobs, 1996; Coltheart et al., 2001; Perry et al., 2007; Grainger and Ziegler, 2008; Hofmann et al., 2011). Importantly, low-performing older adults who were classified based on their results in a sentence comprehension task showed a stronger age-related decline already at the most fundamental level of visual word recognition compared to high-performing older adults.

At the orthographic processing level, larger estimates for drift rates (*v*) were observed for high-performing older adults compared to young adults and, in turn, drift rates (*v*) of young adults were higher than those of low-performing older adults. Our findings differ from those of previous studies (cf. Ratcliff et al., 2004c; Cohen-Shikora and Balota, 2016; Froehlich and Jacobs, 2016), which reported no age-related effects on orthographic processing. This discrepancy very likely is the result of assigning older participants to two performance groups, as suggested by an additional hierarchical diffusion model analysis of our data: When comparing young adults with all older adults assigned to a single large group we replicated the results of Ratcliff et al. (2004c; see Supplementary Material). So what can account for the differences in speed of information uptake in the present study? Usually it is assumed that life-long exposure to text gives older adults a natural advantage in vocabulary knowledge (Allen et al., 1995; Verhaeghen, 2003). This should in turn lead to a larger and better organized orthographic lexicon and likely to higher drift rates in older compared to younger adults. Yet, low-performing older adults might show a reduced drift rate compared to high-performing older and young adults because of a less extensive and efficient orthographic lexicon, if their vocabulary knowledge is not as abundant. Low-performing readers tend to rely more on phonological recoding strategies and engage brain regions typically related to orthographic processing to a lesser extent (Jobard et al., 2011). Interactive activation models of reading, such as the Multiple Read-Out Model (MROM; Grainger and Jacobs, 1996) assume the orthographic lexicon to consist of a network of connected word nodes. Words are positively identified in lexical decision as soon as the activation of a single word node or the summed global activation of all word nodes reaches a certain decision criterion. In low-performing older adults of the present study, either initial lexical activation or the global spreading of activation might be affected as characterized by the lower drift rate (*v*) compared to the other two groups of participants. Findings provided by Robert and Mathey (2007) who investigated age-related effects on lexical inhibition point toward both possibilities: inefficient inhibition and activation processes in older adults. Further research using the MROM or alternative computational models to simulate individual word recognition performance (e.g., Ziegler et al., 1998; Jacobs et al., 2003) is necessary to decide this issue.

Results observed at the phonological processing level mirrored findings identified at the sublexical processing level: Highest drift rates (*v*) were found for young adults; drift rates (*v*) for high-performing older adults were higher than those of low-performing older adults. Because phonological processing requires successful encoding of grapheme-phoneme correspondences, it is conceivable that deficits identified at the sublexical processing level further prevail at the phonological processing level. However, phonological processing is not restricted to decoding simple spelling to sound correspondences of single letters, but also applies to successful whole word recognition (e.g., Coltheart et al., 2001; Perry et al., 2007; Taylor et al., 2012). It is assumed that grapheme-phoneme conversion operates either in a serial manner (e.g., Perry et al., 2007) or depends on the relative level of activation of the corresponding units (Jacobs et al., 1998). In the latter case, both low- and high-performing older adults may show similar deficits in activation mechanisms as proposed at the orthographic processing level for low-performing older adults to varying degrees. In the former case, older adults may experience deficits in temporal sampling. Evidence from speech perception studies suggests that even with a still intact auditory system, older participants persistently are more affected by (auditory) noise and temporal changes in auditory signals (Thornton and Light, 2006; Burke and Shafto, 2008).

At the lexico-semantic processing level, we identified similar results for posterior estimates in the speed of information uptake (*v*) as for orthographic processing. High-performing older adults obtained larger drift rates (*v*) than young adults whose drift rates (*v*) in turn exceeded those of low-performing older adults. These results deviate from the age-related null effects in semantic decision previously reported in the only other diffusion modeling study conducted so far (Spaniol et al., 2006). Again, we believe that this discrepancy is the consequence of dividing the older cohort into two groups which was done to account for the greater variability in performance reported for older adults in our very large sample. To check this, we collapsed the two older groups into a single group and we ran an additional hierarchical diffusion model analysis (see Supplementary Material). Here, we observed smaller drift rates (*v*) for older than for younger readers, a finding that still differs from the data of Spaniol et al. (2006). Yet, we tested a considerably larger sample than these authors, and older adults of the present study showed significant differences in reading performance as measured by the German sentence reading test. These performance differences within the older age group at the sentence comprehension level are visible at every processing level of reading, including the lexico-semantic one. Lexico-semantic processing in word recognition is well captured by the Associative Read-Out Model (AROM; Hofmann et al., 2011; Hofmann and Jacobs, 2014) which extends the framework of the MROM by an associative layer simulating long-term associations between words based on their co-occurrence statistics. Due to the strong connection between the orthographic and lexico-semantic layers in the AROM, lower drift rates (*v*) for low performing older adults who already exhibited deficits at the orthographic processing level, might thus just be due to those orthographic deficits. Alternatively or additionally, in low performing older adults, semantic processing itself might be affected. Yet, to what extent the level of activation or the efficiency of activation spreading or inhibition between associated words is affected by age remains an open issue that calls for further research using simulation modeling of individual performances (see above).

## Conclusion

To summarize, our findings based on a very large sample of readers point toward a highly differentiated picture of age-related effects on word recognition and reading. While at the orthographic and lexico-semantic processing levels high-performing older adults outperform younger adults, older adults generally show age-related deficits both at the sublexical and phonological levels. Low-performing older adults are generally slowest in information uptake in all four reading subprocesses. The present results suggest that these age-related disadvantages are rooted in less efficient activation, inhibition and/or temporal sampling processes in older adults. However, concluding this issue requires further studies combining simulation modeling with neurocognitive methods like EEG or fMRI, i.e., both more sophisticated computational models and more constraining data (e.g., Braun et al., 2006; Hofmann and Jacobs, 2014). Although cross-sectional studies are somewhat limited to distinguish between cohort and true aging effects, we still believe that the present findings contribute substantially to research on aging, because this is the first study to systematically investigate age-related effects on all four basic subprocesses of reading within one very large sample. Since these subprocesses do not operate in an isolated manner but are highly intertwined, a major challenge for future research is to investigate the proposed mechanisms underlying changes in subprocesses over the life-span together with age-related effects on the reading network as a whole. Apart from neurocognitive methods and models, such future research should strive to include more natural reading tasks and texts (Willems and Jacobs, 2016) that may give an advantage to older readers compared to the basic speeded decision tasks used in most of the literature mentioned here.

## Author Contributions

EF, JZ, MB, UL, HH, and AJ contributed to the conception and design of the study. Data was analyzed by EF and interpreted by EF and JL. EF, JL, and AJ drafted the manuscript. It was revised by JZ, MB, UL, and HH. All authors approve the final version of this article and agree on being accountable for all aspects of this work.

## Conflict of Interest Statement

The authors declare that the research was conducted in the absence of any commercial or financial relationships that could be construed as a potential conflict of interest.
