# Supplementary material for: Drifting through Basic Subprocesses of Reading: A Hierarchical Diffusion Model Analysis of Age Effects on Visual Word Recognition
Source: Front Psychol. 2016 Nov 25;7:1863. doi: 10.3389/fpsyg.2016.01863 (PMC5122734; doi:10.3389/fpsyg.2016.01863)
Supplement: Supplementary file 1 [file Data_Sheet_1.docx]

Supplementary Material

Drifting through basic subprocesses of reading: A hierarchical diffusion model analysis of age effects on visual word recognition

Eva Froehlich^1,2,3*^, Johanna Liebig^1,2,3^, Johannes C. Ziegler^4^, Mario Braun^5^, Ulman Lindenberger^6^, Hauke R. Heekeren^1,2,3^, Arthur M. Jacobs^1,2,3^

^1^Department of Education and Psychology, Freie Universität Berlin, Berlin, Germany

^2^Dahlem Institute for Neuroimaging of Emotion, Freie Universität Berlin, Berlin, Germany,

^3^Center for Cognitive Neuroscience, Freie Universität Berlin, Berlin, Germany

^4^Aix-Marseille Université and Centre National de la Recherche Scientifique, Marseille, France

^5^Centre for Cognitive Neuroscience, Universität Salzburg, Salzburg, Austria

^6^Max Planck Institute for Human Development, Berlin, Germany

*** Correspondence:** Eva Fröhlich: eva.froehlich@fu-berlin.de

**Supplementary Table S1.** Mean item characteristics and standard deviations (*SD*) for the single item reading tasks

|  | LIT | |  | LDT | |  | PDT | |  | SDT | |
| --- | --- | --- | --- | --- | --- | --- | --- | --- | --- | --- | --- |
|  | Target | Non-target |  | Target | Non-target |  | Target | Non-target |  | Target | Non-target |
| Length | 5.00  (.00) | 5.00  (.00) |  | 4.53  (.51) | 4.48  (.51) |  | 4.45  (.50) | 4.43  (.50) |  | 4.50  (.51) | 4.53  (.51) |
| WF | -  - | -  - |  | 1.31  (.82) | 1.41  (.83) |  | 1.37  (.75) | 1.33  (.93) |  | 1.27  (1.03) | 1.22  (1.10) |
| BF | 3.89  (.74) | 3.81  (.69) |  | 4.42  (.27) | 4.43  (.27) |  | 4.37  (.31) | 4.35  (.28) |  | 4.42  (.24) | 4.41  (.25) |
| ON | -  - | -  - |  | 23.4  (14.1) | 20.2  (13.6) |  | 20.4  (12.6) | 19.4  (16.1) |  | 24.4  (14.9) | 23.8  (10.7) |

# *Note*. LIT = letter identification task; LDT = lexical decision task; PDT = phonological decision task; SDT = semantic decision task; Length = number of letters; WF = normalized lemma frequency of the word or base word (PDT); BF = bigram frequency; ON = orthographic neighborhood density.

**Additional hierarchical diffusion model analyses on orthographic and lexico-semantic processing with older adults being binned into one large group (N = 1,423)**

**Model set-up.** For the additional analyses, we estimated the posterior distributions of a total of 12 parameters across the lexical and lexico-semantic decision task: four non-decision time parameters (*t*), four threshold parameters (*a*) with the upper threshold being the correct response and the lower threshold being the incorrect response and four drift rate parameters (*v*; for *t, a,* and *v* one for each age group within each of the two tasks).

**Assessment of convergence and model fit.** No drifts nor large jumps were observed when inspecting the traces of the posterior distributions for each of the two models separately. Furthermore, we observed no parameter values above 1.02 within the R-hat statistic which indicates successful convergence for both the orthographic and the lexico-semantic model. The models fitted the data well as correlations between model RT quantiles and the observed data was *r* = .94 in the lexical decision task, and *r* = .96 in the semantic decision task (Supplementary Figure S1).

**Model parameter analysis of posterior estimates.** Older adults obtained larger posterior estimates for non-decision time (*t*) and decision threshold (*a*) than young adults with a probability of 1, as assessed via Bayesian hypothesis testing, for both the lexical decision and the semantic decision task (Supplementary Figure S2). Estimates for drift rates (*v*) were smaller for older compared to young adults in the semantic decision task (again exhibiting a probability of 1). Within the lexical decision task, however, the probability of drift rates being larger for younger than for older adults was only .50.

#
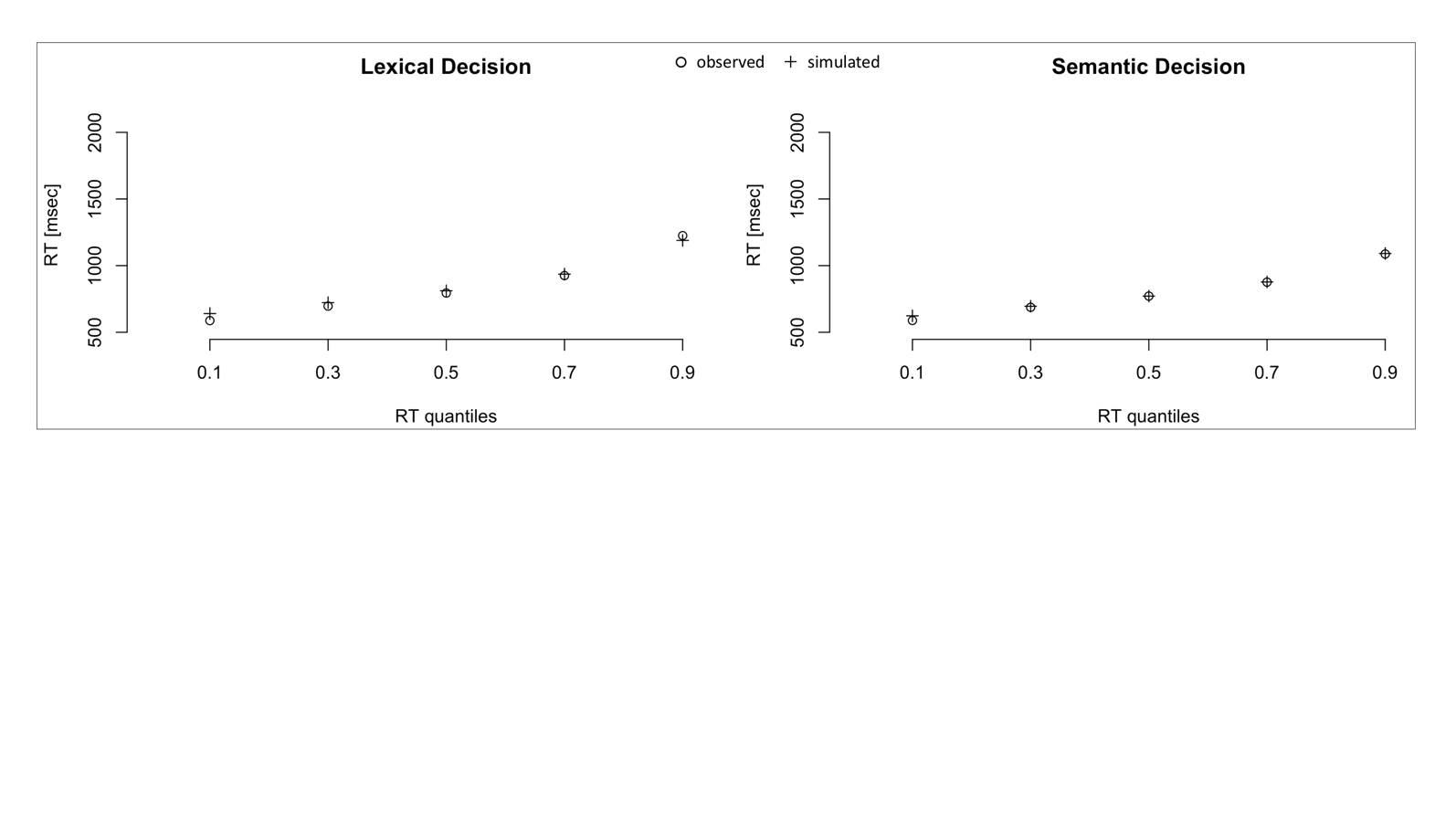
Supplementary Figures

# Figure S1. Plot of RT quantiles (0.1, 0.3, 0.5, 0.7, 0.9) for correct responses based on observed and simulated data

#
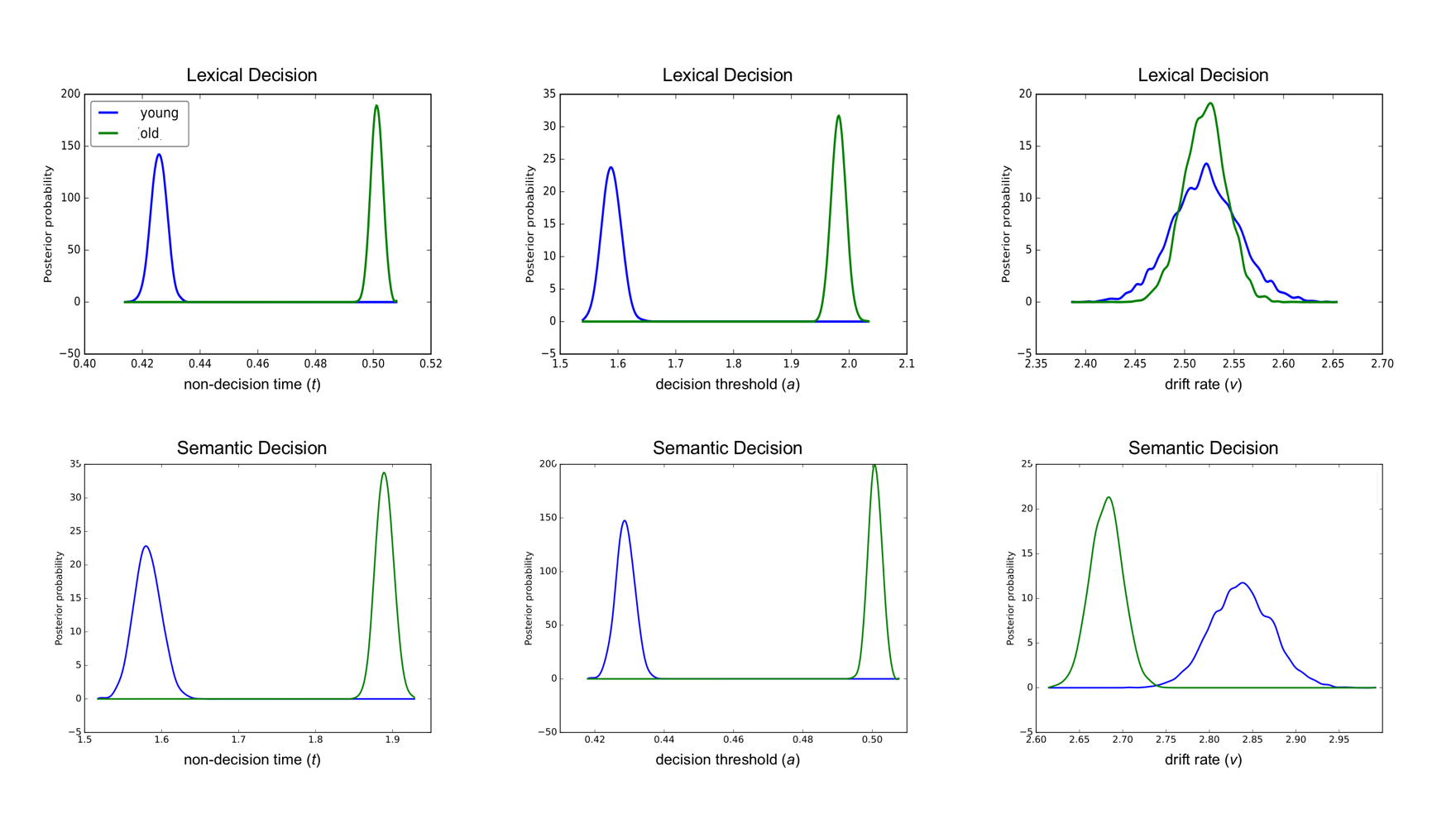


**Figure S2*.*** Posterior density plots of group means of the parameters non-decision time *t* (first column), decision threshold *a* (second column) and drift rate *v* (third column)
